# Supplementary material for: Drosulfakinin signaling modulates female sexual receptivity in Drosophila
Source: eLife. 2022 Apr 27;11:e76025. doi: 10.7554/eLife.76025 (PMC9045819; doi:10.7554/eLife.76025)
Supplement: Supplementary file 1. — The numbers were shown in the table are the number of pairs that copulated within the 30 min divided by the number of total tested pairs. None of females successfully copulated in mated females and very young females, except one mated female successfully copulated with the genotype of UAS-dTrpA1/+ at 29°C. [file elife-76025-supp1.docx]

Table S1. Activation of DSK neurons did not change receptivity in mated females and very young females, related to Figure2.

|  | *UAS-dTrpA1/UAS-dTrpA1;*  *Dsk^GAL4^/Dsk^GAL4^* | *UAS-dTrpA1/+* | *Dsk^GAL4^/+* |
| --- | --- | --- | --- |
| Mated female, 21°C | 0/61 | 0/63 | 0/72 |
| Mated female, 29°C | 0/67 | 1/70 | 0/68 |
| 12-18hr Virgin, 21°C | 0/75 | 0/69 | 0/70 |
| 12-18hr Virgin, 29°C | 0/72 | 0/75 | 0/69 |

The numbers were shown in the table are the number of pairs that copulated within the 30 min divided by the number of total tested pairs. None of females successfully copulated in mated females and every young females, except one mated female successfully copulated with the genotype of *UAS-dTrpA1/+* at 29°C.
