# Supplementary material for: Drosulfakinin signaling modulates female sexual receptivity in Drosophila
Source: eLife. 2022 Apr 27;11:e76025. doi: 10.7554/eLife.76025 (PMC9045819; doi:10.7554/eLife.76025)
Supplement: Supplementary file 2. [file elife-76025-supp2.docx]

Table S2. Synaptic connections identified by EM reconstruction.

|  | post  (Cell ID) | | | |
| --- | --- | --- | --- | --- |
| pre  (Cell ID) | DSKMP1A_R (1135837629) | DSKMP1B_R (1352077058) | DSKMP1B_L (1011184205) | DSKMP3_R (327937328) |
| pC1a_R (5813046951) | 0 | 0 | 1 | 0 |
| pC1a_L (359744514) | 0 | 5 | 0 | 0 |
| pC1b_R (267214250) | 0 | 15 | 0 | 1 |
| pC1c_L (550319575) | 0 | 2 | 2 | 0 |
| pC1c_R (267551639) | 1 | 84 | 1 | 1 |
| pC1d_R (5813063587) | 2 | 2 | 0 | 0 |
| pC1e_R (514850616) | 0 | 2 | 0 | 0 |

Number of synaptic connections identified between pC1 neurons and DSK neurons. X_R and X_L indicate right- and left-hemisphere X cells (X indicates pC1 cells or DSK cells).
